# Physical durability: are bed nets getting any stronger?

**DOI:** 10.1186/s12936-023-04832-8

**Published:** 2024-01-13

**Authors:** Amy Wheldrake, Estelle Guillemois, Hamidreza Arouni, Vera Chetty, Edoardo Zappa, Stephen J. Russell

**Affiliations:** Nonwovens Innovation & Research Institute Ltd, Innovation House, Gibraltar Island Rd, Leeds, West Yorkshire LS10 1RJ UK

**Keywords:** Long-lasting insecticidal mosquito nets, Resistance to damage, Physical integrity, Durability

## Abstract

**Background:**

For at least a decade, concerns have been raised about the physical durability of insecticide-treated nets (ITNs) and their ability to remain in good condition for at least three years. To discover if the resistance to damage (RD) of ITNs has improved or not, the RD scores of ITNs sampled in 2013 and 2020 were compared.

**Methods:**

The RD scores and disaggregated textile performance data for nine ITNs recommended by the WHO pesticide evaluation scheme (WHOPES) measured in 2013 were compared with WHO-prequalified ITNs sampled in 2020. This included assessment of newer ITNs not available in 2013, to determine the extent to which product development has led to performance improvements across all available ITNs in the intervening years.

**Results:**

The resistance to damage of ITNs has not generally improved from 2013 to 2020, and in some cases performance is worse. The average RD score of comparable ITNs brands decreased from 40 in 2013 to 36 in 2020. Of the nets available in 2020, only two of the twenty-four ITN products tested achieved an RD score of > 50, while six ITNs had very low RD scores of < 30, highlighting a serious inherent, and literal weakness in many WHO-prequalified ITNs.

**Conclusions:**

The long-term physical durability of ITN products cannot be expected to improve while their resistance to damage remains so low, and major upgrades to the performance standards of textile materials used to make ITNs, as well as incentives to develop stronger ones are urgently required.

## Background

For decades, insecticide-treated nets (ITNs) have played an important role in preventing malaria cases and deaths in malaria-endemic countries. Between 2000 and 2015, the malaria case incidence declined by 27% owing mostly to mass distribution campaigns of ITNs in malaria-risk countries [1, 2]. The malaria case incidence slightly increased due to disruption caused by the Coronavirus disease (COVID-19) pandemic in 2020. However, while ITNs are still being widely distributed in endemic countries every three years, recent malaria case incidence has not substantially reduced [3].

Various explanations may be posited to explain the lack of a downward trend in malaria case incidence, but one contributory factor could be related to the lack of physical durability provided by modern ITN products. Clearly, if nets are insufficiently robust and quickly accumulate holes that undermine their ability to provide physical protection, they can no longer perform their basic function. Field studies have repeatedly reported loss of ITN physical integrity within two years post-distribution, with many ITNs being so badly damaged, they are discarded [4].

Over the same period there has been a lack of performance standards in product specifications relating to the physical durability of nets, meaning manufacturers have not been given appropriate targets to address. This has created a disconnect between the engineering design of ITN products and their expected performance in the field. Meanwhile, the weighted average price for ITNs sharply decreased from USD 4.20 per ITN in 2011, down to USD 1.88 per ITN in 2019 due to greater cost transparency, volume, industry consultation and synchronised demand [5]. This raises the question as to whether the physical durability of ITNs has also been impacted by product design changes, in response to price pressures. With so much emphasis being placed on long-term insecticidal functionality, the critical role the net textile plays in providing physical protection has been given insufficient attention. If the textile forming the net is too weak to withstand forces it is exposed to during use, large holes can be expected to form rapidly, compromising the ITN’s ability to provide physical protection, regardless of the insecticide’s bioefficacy. The physical durability of an ITN therefore depends on its inherent strength and ability to resist damage, which is governed by textile properties, as well as how carefully it is used following distribution.

The inherent Resistance to Damage (RD) of any ITN can be measured before it is distributed. It depends on the mechanical properties of the textile used to manufacture the net and can be determined by a suite of four laboratory textile tests, in which fabric properties linked to damage mechanisms observed in the field are measured under controlled conditions. Each of these properties is a measure of the ability of the net to withstand the mechanical forces it will be exposed to during normal household use.

The RD methodology introduced by Wheldrake et al. [6–9] has also highlighted marked differences in the performance of existing WHO prequalified ITNs. The RD score characterises the inherent resistance to damage of an ITN, and is measured on a 0–100 scale, where a RD score of 100 indicates the highest performance. Knowing the RD score of an ITN product before it is distributed is, therefore, a useful indicator of its inherent robustness and ability to retain physical integrity during normal use. A link between the RD performance and survivorship has been confirmed by Kilian et al. who reported a correlation between RD scores and actual ITN performance in the field, where RD scores measured in the laboratory of > 50, resulted in substantially longer service life in the field [10].

Given the ongoing concerns about the physical durability of ITNs, the purpose of this study was to determine if there have been any changes in the inherent resistance to damage of ITNs from 2013 to 2020. Test results for WHOPES-recommended (2013) and WHO-prequalified (2020) branded ITNs sampled in 2013 and 2020, respectively, were compared based on the textile testing methodology of Wheldrake et al*.* [6–9]. The study involved many different branded ITNs, including WHO-PQ qualified nets available in 2020, and aimed to identify any trends or changes in performance over the seven-year period.

## Methods

### ITN samples

Existing data for nine WHOPES-recommended ITNs measured in 2013 by Wheldrake et al*.* [7] was compared with primary data for twenty-four WHO-prequalified ITNs sampled in 2020. This included ITN products that were available both in 2013 and 2020, as well as newer products that were not available in 2013. The purpose of including both was not just to compare performance changes in the same products, but also to determine the extent to which product development has led to performance improvements across all available ITNs. The ITNs were made by different suppliers: Vestergaard Frandsen; Tianjin Yorkool International Trading Co., Ltd; Bayer industry Co., Ltd; V.K.A Polymers Pvt Ltd; Sumitomo chemical; BASF Agro B.V. Arnhem; Disease Control Technologies; Tana netting FZ; Shobikaa Impex Private Ltd. and Moon Netting.

**Fig. 1:**
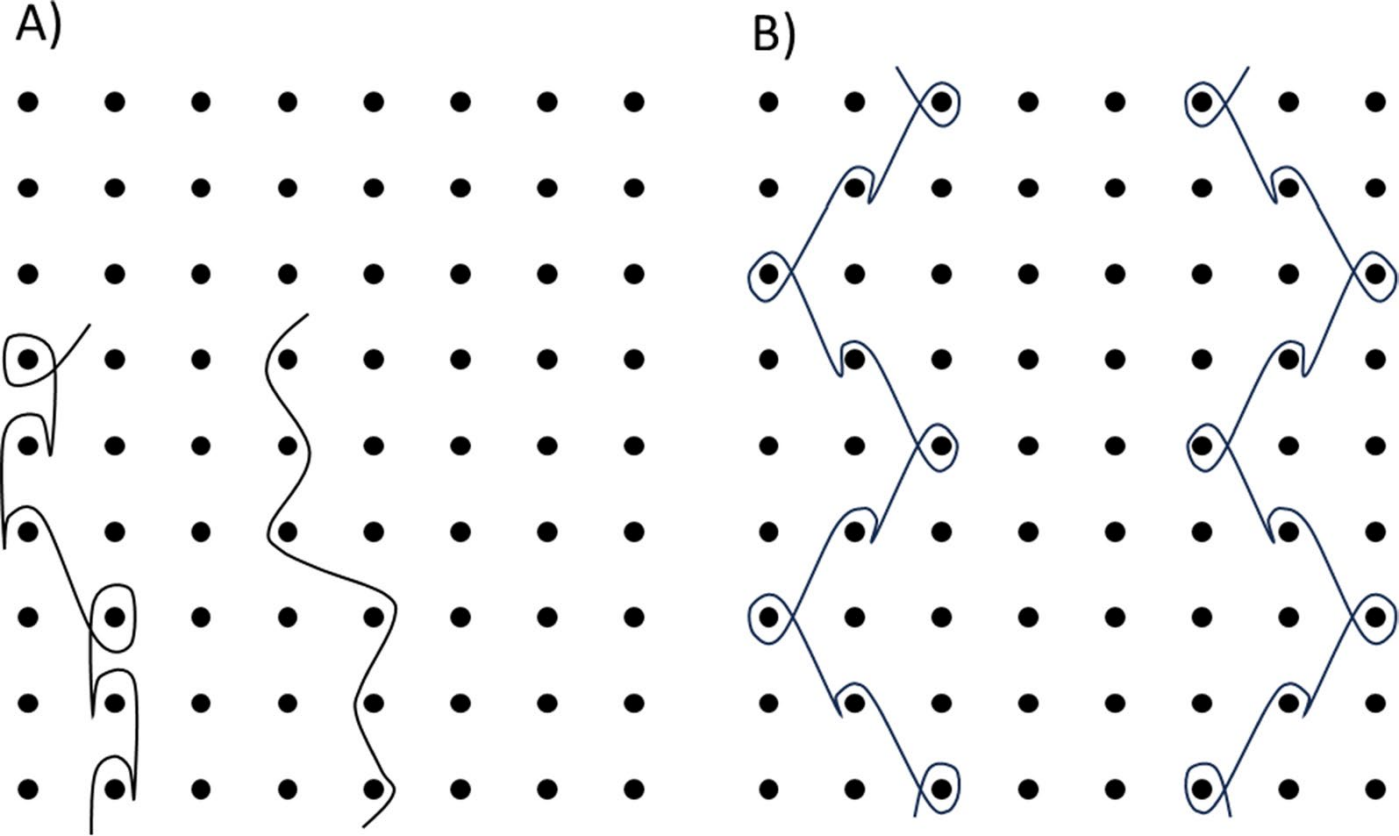
Tulle (**A**) and Traverse (**B**) knitting patterns

Comparison was possible between several of the ITNs available in 2020 and 2013 because in most cases they were produced by the same supplier and were identically branded. Herein, the ITNs are anonymously labelled, and general product specifications are given in Table 1 and Fig. 1, including their principal polymer compositions (as indicated on the product packaging), abbreviated as follows: high density polyethylene (HDPE); polyethylene terephthalate (PET) and polyethylene (PE). The area densities of the ITNs ranged from 27 g m^−2^ to 52 g m^−2^. Ownership of the Net U product transferred to another company between 2013 and 2020 so this lineage was reflected in the coding, even though the ITN specification also changed.

**Table 1:** ITN products sampled and tested in 2013 and 2020

Net	Filament type	Knitting pattern	Year	Area density (gˑm^−2^)	Mesh count (holesˑin^−2^)	Linear density (Denier)
Net A	HDPE monofilament	Tulle	2020	37	108	130
**Net B**	**HDPE monofilament**	**Tulle**	**2013** **2020**	**50** **50**	**164** **164**	**150** **150**
**Net C**	**PET multifilament**	**Traverse**	**2013** **2020**	**42** **43**	**156** **164**	**100** **100**
Net D	PET multifilament	Traverse	2020	42	155	100
Net E	PE monofilament	Tulle	2020	47	138	120
Net F	PE monofilament	Tulle	2020	36	130	120
Net G	PE monofilament	Tulle	2020	41	76	130
**Net H**	**HDPE monofilament**	**Tulle**	**2013** **2020**	**50** **50**	**132** **148**	**150** **150**
Net I	PE monofilament	Tulle	2020	39	128	120
Net J	PET multifilament	Traverse	2020	27	142	75
Net K	PET multifilament	Traverse	2020	43	162	100
**Net L**	**PET multifilament**	**Traverse**	**2013** **2020**	**42** **39**	**156** **165**	**100** **100**
Net M	PET multifilament	Traverse	2020	43	96	150
**Net N**	**PET multifilament**	**Traverse**	**2013** **2020**	**41** **41**	**156** **176**	**100** **100**
Net O	PET multifilament	Traverse	2020	32	157	75
Net P	PET multifilament	Traverse	2020	42	168	100
**Net Q**	**PET multifilament**	**Traverse**	**2013** **2020**	**42** **44**	**156** **186**	**100** **100**
Net R	PET multifilament	Traverse	2020	28	154	75
Net S	PET multifilament	Traverse	2020	45	96	150
Net T	PE monofilament	Tulle	2020	52	145	150
**Net U**	**PET multifilament** **PE monofilament**	**Traverse** **Tulle**	**2013** **2020**	**42** **37**	**156** **100**	**150** **120**
Net V	PE monofilament	Tulle	2020	32	132	130
**Net W**	**PE monofilament**	**Tulle**	**2013** **2020**	**43** **29**	**75** **72**	**150** **150**
**Net X**	**PE monofilament**	**Tulle**	**2013** **2020**	**43** **33**	**80** **111**	**150** **135**

Nets highlighted in bold were compared in 2013 and 2020

### Laboratory testing of ITNs

All ITN samples were tested in accordance with RD methodology, which involves four textile tests each of which reflects different mechanisms of damage that nets are exposed to in the field [6]. For each ITN, the sample preparation methods, test procedures and number of replicates recommended by Wheldrake et al*.* [7, 9] were followed to obtain data for the bursting strength, snag strength, abrasion resistance and resistance to hole enlargement. For each ITN, three separate net samples were analysed with five measurements per sample. The sampling method also accounts for those products with different fabrics forming the roof and side panels.

### Resistance to Damage (RD) scores

The RD methodology developed by Wheldrake et al*.* [8] enables a single performance metric to be obtained by aggregating the textile testing data for snag strength, abrasion resistance, bursting strength and hole enlargement resistance, together with human factors (to obtain aspirational performance values). The RD score characterises the inherent resistance to damage of the ITN on a RD = 0–100 scale, where RD = 100 represents the highest performance as shown in Eq. 1[8].

1 $${\text{RD}} = \left( { \frac{{\lambda_{B} }}{{\eta_{B} }} \times \frac{100}{4}} \right) + \left( { \frac{{\lambda_{S} }}{{\eta_{S} }} \times \frac{100}{4}} \right) + \left( { \frac{{\lambda_{A} }}{{\eta_{A} }} \times \frac{100}{4}} \right) + \frac{{\sigma_{H} }}{4}$$

where:

RD = Resistance to damage.

${\lambda }_{B}$= Actual bursting strength (kPa).

$\eta_{B}$ = Aspirational bursting strength (kPa) 

${\lambda }_{S}$ = Actual snag strength (N).

$\eta_{S}$ = Aspirational snag strength (N).

${\lambda }_{A}$= Actual abrasion resistance strength (number of rubs).

$\eta_{A}$ = Aspirational abrasion resistance (number of rubs).

σ_H_ = Hole enlargement resistance.

## Results

To enable comparison between 2013 and 2020, the mean data for each physical property associated with the RD methodology is reported, together with the aggregated RD scores for all ITNs.

### Bursting strength

**Fig. 2:**
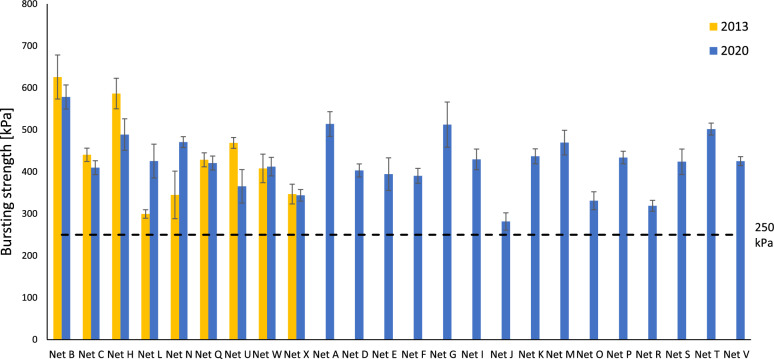
Mean bursting strength values for ITNs in 2013 and 2020. Error bars correspond to standard deviation (n = 15)−−250 kPa threshold is the bursting strength requirement set by WHO

Although bursting strength alone is a poor predictor of physical integrity and resistance to hole formation [9], it is a useful measure when aggregated with other textile mechanical property data, and measurements form part of the RD score. Figure 2 reveals that in 2013 the mean bursting strength of ITNs ranged from 299 kPa to 626 kPa, and in 2020, from 281 kPa to 578 kPa. Except for Net N and Net L which performed significantly better (p < 0.05) (+ 37% and + 42%, respectively), the ITNs in 2020 performed similarly, or worse compared to 2013. Nets B, C, H and U had significantly lower bursting strengths (p < 0.05) in 2020 compared to 2013 (− 8%, − 7%, − 17%, − 22% respectively) whereas Nets Q, W and X exhibited similar bursting strength in 2020 and in 2013 (p > 0.05). Note that all ITNs passed the long-standing bursting strength requirement set by WHO of 250 kPa, with most achieving values > 400 kPa in 2020. This reflects the fact that each ITN product has its own bursting strength requirements and specifications, which can be substantially higher than 250 kPa.

### Snag strength

**Fig. 3:**
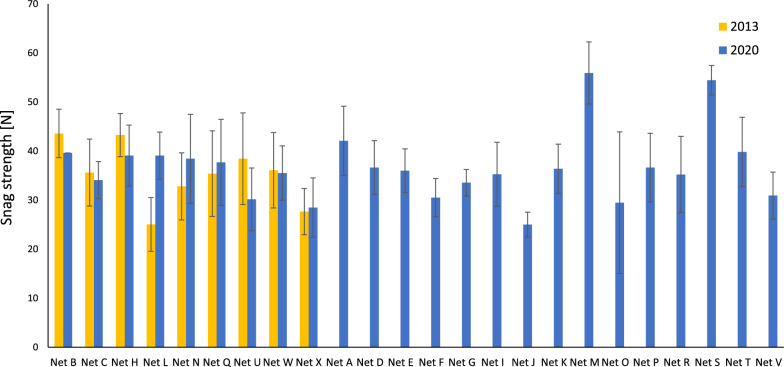
Mean snag strength values for ITNs in 2013 and 2020. Error bars correspond to standard deviation (n = 30)

Snagging is the most frequently encountered form of mechanical damage in ITNs retrieved from the field and is an inherent weakness of lightweight knitted fabrics [11]. Figure 3 illustrates marked differences in the mean snag strengths across ITNs (2013 and 2020) from as low as 25N to 56N. Nets L and N performed significantly better in 2020 (p < 0.05) than in 2013 (+ 56% and + 17% respectively), while the snag strength of Net B, H and U significantly decreased by approximately − 9, − 10 and − 22%, respectively in 2020 compared to 2013 (p < 0.05). In respect of Net U (2020), this is likely to be attributable to a decrease in the yarn linear density and the area density of the fabric compared to 2013.

### Abrasion resistance

**Fig. 4:**
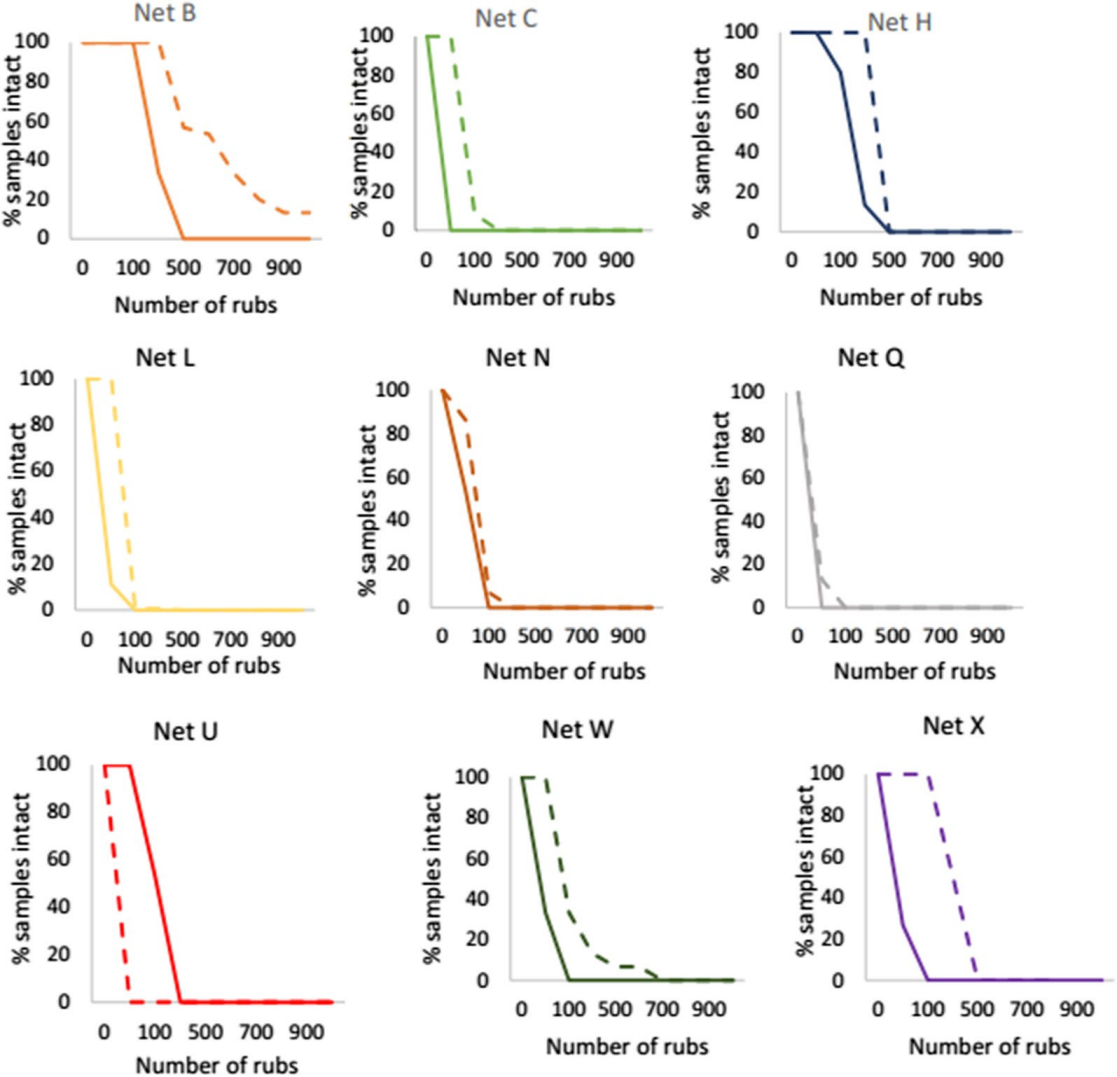
Abrasion resistance of ITNs in 2013 (dotted line) and 2020 (solid line)

Figure 4 compares the abrasion resistance of ITNs in 2013 and 2020, specifically the rate at which nets failed as the number of rubs (abrasion cycles) applied to the net increased. Except for Net U, the abrasion resistance of the 2020 ITN samples was similar, or worse than the 2013 performance.

### Hole enlargement resistance

**Fig. 5:**
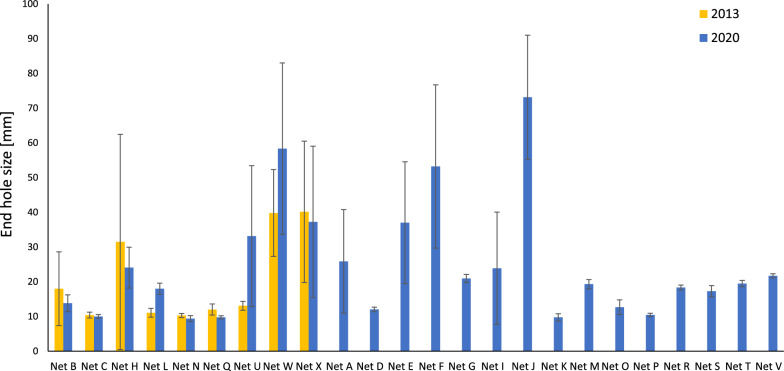
Mean hole enlargement (end hole size) for ITNs in 2013 and 2020. Error bars correspond to standard deviation (n = 15)

A comparison of the hole enlargement resistance of ITNs in 2013 and 2020 is shown in Fig. 5. A significant decrease in the performance of Net L, Net U and Net W was observed between 2013 and 2020 (p < 0.05) with an increase in the end hole size of + 63%, + 154% and + 47% respectively. However, Net N and Net Q showed significantly improved performance in 2020 (p < 0.05) with reductions in the end hole size of -8% -19% respectively. Large differences in hole enlargement resistance were evident between different ITN products, with the worst performing ITNs generally being made from polyethylene (PE) monofilament warp knitted tulle fabrics. Across all nets, end hole sizes ranged from only 9.4 mm for Net N (very resistant to hole enlargement), to 73 mm for Net J, which is so large it is likely to completely undermine the ITN’s ability to provide long-term physical protection.

### Resistance to damage (RD) scores for ITNs

**Fig. 6:**
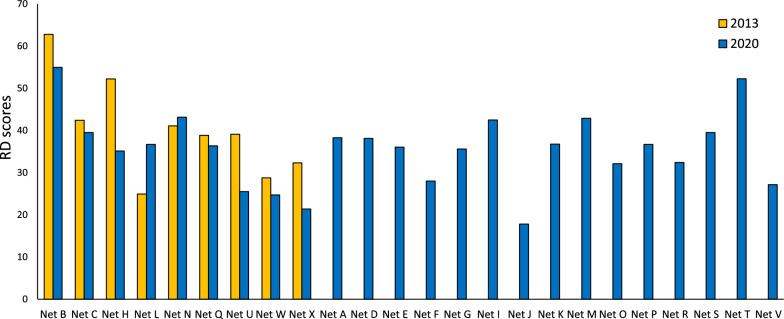
Resistance to Damage (RD) scores for ITNs in 2013 and 2020

A comparison of RD scores for ITNs in 2013 and 2020 are reported in Fig. 6. These scores highlight marked differences in the performance of different products. According to RD methodology, the performance of ITNs should be achieving RD > 50, and ideally approaching RD = 100. Comparing the results from 2013 and 2020, a reduction in the RD value was observed for Net B, Net C net Q, Net H, Net U, Net W and Net X of -12%, -7%, -6%, -33%, -35%, -14% and -34%, respectively. Only Net N and Net L showed improved RD values between 2013 and 2020 of + 5 and + 47%, respectively.

In 2020, only Net B and Net T achieved RD scores > 50. Apart from Net N and Net L, the RD scores of ITNs in 2020 were lower than in 2013. Concerningly, in 2020, six out of twenty-four WHO-recommended ITNs (25%) produced very low RD scores of < 30, making them highly susceptible to mechanical damage in the field and compromising their ability to provide long-term physical durability in the field.

**Fig. 7:**
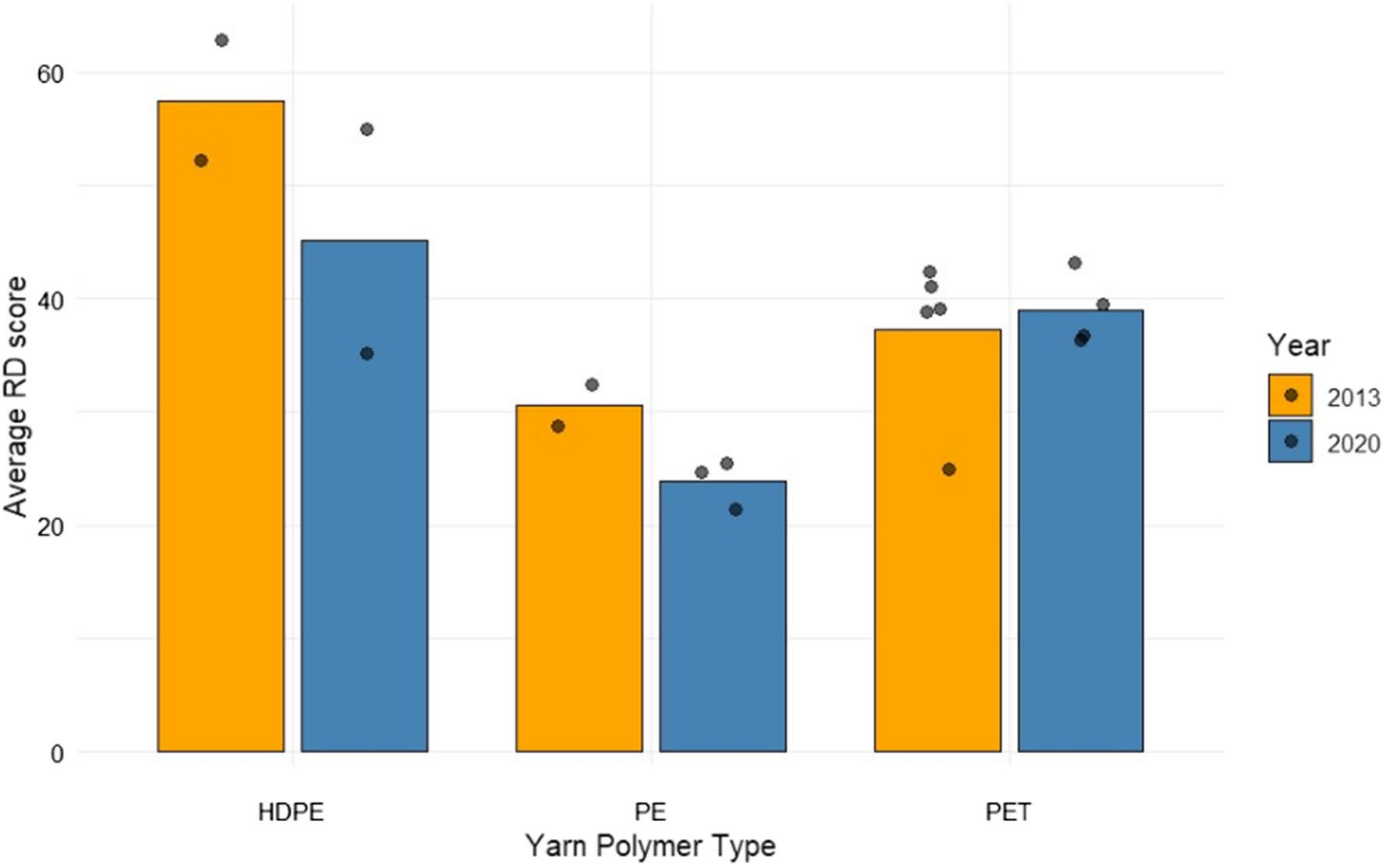
Resistance to Damage (RD) scores for ITNs in 2013 and 2020 grouped by yarn material (polymer) and year. Where, HDPE is high density polyethylene; PE is polyethylene, and PET is polyethylene terephthalate. Data points overlapped on the graph to show variability

The average RD score of all comparable ITNs brands (excluding Net U, which was subject to a known specification change in the intervening years) decreased from 40 in 2013 to 36 in 2020. Figure 7 compares the average RD scores of nets in 2013 and 2020 and indicates reductions from 57 to 44 (for HDPE) and from 31 to 24 (for PE). However, the average RD scores for PET nets did not follow a similar trend. Note that there are also differences in the type of yarn construction (mono and multifilament) as well as knitting patterns, which means caution is needed in making generalised conclusions about the performance of nets made from different polymer types. Certainly, it is evident that there has been no major improvement in average RD scores in the seven-year period for any of the nets grouped by polymer type.

## Discussion

Over the last decade, field study evaluations of ITNs have repeatedly reported unsatisfactory performance in terms of physical integrity and survivorship, due to nets becoming too torn or damaged to provide long-term physical protection for at least three years. Whilst progress has been made in developing new insecticidal formulations, relatively little attention has been paid to the importance of the net textile itself, even though it plays a vital role in providing physical barrier protection and substantial physical damage could result in the net being disposed of [2]. Apart from bursting strength, no pre-assessment of the inherent ability of an ITN to resist damage is normally required prior to its approval and distribution, even though holes and tears are generally regarded as inevitable.

ITNs with inherently high resistance to damage can be expected to last longer in the field, promoting improved survivorship, and this basic hypothesis has been confirmed by Kilian et al. [10] who demonstrated that existing ITNs achieving RD scores > 50, improved survivorship in the field by approximately seven months. However, as evidenced in for example Fig. 5 (hole enlargement), some ITNs resist damage so poorly that their ability to provide long-term physical protection is highly questionable. The fact that existing ITN products are not ‘the same’ in terms of their resistance to damage may also partly explain why the physical durability of ITNs generally appears to be so variable.

The disconnect between the performance standards ITNs need to meet in the lab, and their required performance in the field, is in stark contrast to the insecticide, which is subject to thorough laboratory testing, as well as semi- and full field testing to ensure bioefficacy before use. Elsewhere, textiles used to make products for protecting people from potential harm, such as personal protective equipment (PPE) are subject to much stricter performance standards in the laboratory than ITNs, to ensure they are fit for purpose. ITN textiles need to be more thoroughly tested in the laboratory, based on appropriate performance standards, measuring properties that are relevant to the required performance in the field. In this regard, it is positive that WHO PQ-Vector Control team has made constructive steps to consider adoption of additional textile performance standards to drive improvements in the physical durability of ITNs.

The pressing need for better ITN performance standards related to physical durability is highlighted by comparing the inherent resistance to damage data for ITNs sampled in 2013 and 2020, which reveals that the performance of many ITN products has not improved in seven years (Figs. 2, 3, 4, 5 and 6). In 2020, only two of the twenty-four ITNs examined achieved an RD score > 50, while 25% of the ITN products available achieved a very low RD score of < 30 (Fig. 6). The ITNs with RD scores > 50 (Net B and T) were made from fabrics having relatively high area densities, mesh counts and linear density.

By contrast in 2013, six of the sixteen WHOPES-recommended ITNs available, achieved RD values of > 50 [7]. The generally lower RD values observed in 2020 compared to 2013 is also highlighted when comparing ITNs in terms of their polymer composition (Fig. 7), although comparisons between nets made from HDPE, PE and PET are not straightforward because the knitting pattern is not consistent between products.

Low RD values are likely to increase the vulnerability of ITNs to physical damage in the field, such that they will be prone to accumulating holes more quickly, limiting their long-term service life. Given the inherent weakness of current ITNs, particular focus is needed on improving abrasion resistance (Fig. 4), snag strength (Fig. 3) and hole enlargement resistance (Fig. 5), to markedly increase overall resistance to damage. This is likely to require major upgrades to the specifications of textile fabrics used to manufacture ITNs, including but not limited to polymer grades, filament linear density, knitting pattern, as well as basis weight (g/m^2^). Of course, this will have cost implications, but these should be balanced against resulting increases in the reliability and value of ITNs in providing longer service life. At the same time, user perceptions and experience are key to ensuring on-going product acceptance and so the design of more durable ITNs should also consider factors such as the aesthetics and thermophysical comfort of products.

The availability of robust, more damage resistant ITNs is not only a technical issue, but one of market dynamics. The design of more physically durable, longer-lasting ITN products is feasible, but there is little incentive to pursue improvements or innovate given that prices and margins are so low. As evidenced in this study, even though existing WHO Prequalified ITNs are not ‘the same’ in terms of their inherent resistance to damage, there is no recognition of this in procurement policies. Progressive price reductions in ITNs over many years combined with specifications that do not reward quality in terms of product performance standards, are disincentivising innovation and risk stagnating product innovation. Price pressures also incentivise cost-cutting, which is likely to negatively impact product performance. Evidence of changes to product specifications since 2013 (Table 1) leading to a fall in ITN performance are apparent in the present data. For example, the drop in bursting strength (Fig. 2), abrasion resistance (Fig. 4) and snag strength of Net U from 2013 to 2020 is likely to be attributable to the reduced fabric basis weight (-12%), together with decrease in both the mesh count and filament linear density of approximately, -36% and -20%, respectively.

Raising the bar by upgrading textile performance standards for ITNs, combined with better pricing policies could act as a positive incentive for innovation, and drive major improvements in physical durability in the field. Upgraded textile performance standards would also ensure that better performance is built in to ITN products prior to use, instead of physical durability issues being highlighted when it is too late, or after lengthy field studies. Practically, this means implementing additional textile testing requirements and quantitative performance standards for snag strength, abrasion resistance and hole enlargement resistance, alongside bursting strength. It is the implementation of the individual textile testing methods that is key. Aggregating the resulting data to determine RD would be an optional step. Such an approach is also likely to lead to a more cost and time-efficient process for ITN development and evaluation, ensuring ITNs become longer-lasting more quickly, and truly fit for purpose.

## Conclusion

The inherent resistance to damage (RD) of ITNs has not markedly improved in the seven-year period from 2013 to 2020, and the performance of some ITN products has declined. This is likely to be a contributory factor in the high rates of ITN attrition and poor survivorship that have been repeatedly reported in field studies over the last decade. Of the WHO pre-qualified ITNs tested in 2020, only two achieved an RD score > 50, and six scored < 30, indicating a high degree of inherent weakness amongst currently available products. There are also large differences in the performance of existing ITNs, such that they cannot all be considered ‘the same’. More rigorous textile testing of ITN products is required, to provide new performance standards for snag strength, hole enlargement resistance and abrasion resistance to encourage development of more physically durable, longer-lasting ITN products, capable of protecting users more effectively. The resulting metrics would also assist procurers and countries to make informed choices.

## Data Availability

Datasets are available on reasonable request to NIRI, UK.
